# TCAD Device Simulation of All-Polymer Solar Cells for Indoor Applications: Potential for Tandem vs. Single Junction Cells

**DOI:** 10.3390/polym15092217

**Published:** 2023-05-08

**Authors:** Tarek I. Alanazi

**Affiliations:** Department of Physics, College of Science, Northern Border University, Arar 73222, Saudi Arabia; tarek.alanazi@nbu.edu.sa

**Keywords:** all-polymer, tandem, PCE, indoor, TCAD

## Abstract

The utilization of indoor photovoltaics makes it feasible to harvest energy from artificial light sources. Although single-junction indoor photovoltaics have demonstrated exceptional efficacy when using LED lighting, there is still a need for more comprehensive testing of tandem structures. Herein, the first systematic TCAD simulation study on the potential for tandem all-polymer solar cells (all-PSCs) for indoor applications is provided. The presented all-PSCs are based on experimental work in which the top wide bandgap subcell comprises a polymer blend PM7:PIDT, while the bottom narrow bandgap subcell has a polymer blend PM6:PY-IT. Standalone and tandem cells are simulated under AM1.5G solar radiation, and the simulation results are compared with measurements to calibrate the physical models and material parameters revealing PCE values of 10.11%, 16.50%, and 17.58% for the front, rear, and tandem cells, respectively. Next, we assessed the performance characteristics of the three cells under a white LED environment for different color temperatures and light intensities. The results showed a superior performance of the front cell, while a deterioration in the performance was observed for the tandem cell, reflecting in a lower PCE of 16.22% at a color temperature of 2900 K. Thus, an optimized tandem for outdoor applications was not suitable for indoor conditions. In order to alleviate this issue, we propose designing the tandem for indoor lightening by an appropriate choice of thicknesses of the top and bottom absorber layers in order to achieve the current matching point. Reducing the top absorber thickness while slightly increasing the bottom thickness resulted in a higher PCE of 27.80% at 2900 K.

## 1. Introduction

Photovoltaic (PV) technologies are poised to play a vital role in the transition to more sustainable energy solutions [1], with crystalline Si currently serving as the leading semiconducting material. Different Si-based structures were researched, where some have relatively higher costs [2,3], while others have lower efficiencies [4,5,6,7]. On the other hand, thin-film solar cells represent the latest advancement in PV technology. An ideal photoactive material for use in solar cells must meet several key requirements, including high optical absorption, long diffusion lengths, and the ability to form a decent electronic junction (homo/hetero/Schottky) with appropriately well-matched materials [8,9,10]. In this context, all-polymer solar cells (all-PSCs) represent a highly competitive PV technology with high efficiency and low cost. These solar cells possess a high absorption coefficient, enabling less material usage compared to c-Si technology. All-PSCs offer priority in terms of the cost of production, combining lower energy requirements for manufacturing with superb thermal stability and mechanical flexibility [11,12,13]. In recent years, the power conversion efficiency (PCE) of all-PSCs has progressively boosted from less than 10% to about 18% [14,15,16].

Research efforts are currently concentrating on multi-junction solar cells, where thermalization losses can be minimized, assisting higher PCEs of solar cells. In this regard, the double-junction (tandem) solar cell is the simplest device model, which presents a favorable solution for overcoming the Schottky–Queisser efficiency limit of single-junction PV devices. Tandem solar cells comprise two solar cell technologies arranged on top of each other, where the solar spectrum is split between them [17,18]. The development of tandem all-PSCs is under intensive research to combine the benefits of polymers and the higher PCEs achieved through tandem structures [16,19,20,21]. However, one of the great challenges in designing tandem polymer solar cells is balancing the short-circuit current densities of the two sub-cells. This is because the current of each sub-cell is determined by the amount of light it absorbs, and if the absorption of one sub-cell is too high relative to the other, it can lead to an imbalance in the short circuit current and reduced overall efficiency.

In [19], the authors presented the first report on all-polymer tandem utilizing the same sub-cells based on P2F-DO:N2200. A PCE of about 6.70% was achieved for the tandem structure ITO/ZnO/P2F-DO:N2200/PEDOT:PSS/ZnO/P2F-DO:N2200/MoO_3_/Al, demonstrating the potential of tandem all-PSCs in the field of PVs [19]. Further, a developed homojunction tandem all-PSCs that exhibit both a record PCE of over 11% and remarkable stability has been presented for a tandem structure ITO/PEDOT:PSS/PTzBI-Si:N2200/PNDIT-F3N:PEI/PEDOT:PSS/PTzBI-Si:N2200/PNDIT-F3N/Ag [20]. In [21], a multiscale simulation approach was employed to optimize the cell configurations in polymer-based tandem cells employing P3HT:PCBM and PCPDTBT:PCBM. By applying the simulation approach for a tandem device comprised of ITO/PEDOT:PSS/P3HT:PCBM/TiO_2_/PEDOT:PSS/PCPDTBT:PCBM/TiO_2_/Al, a PCE of 10% was obtained [21]. Recently, researchers achieved high PCE in tandem all-PSCs by using two polymerized small molecular acceptors (PSMAs), where the top subcell was composed of a wide bandgap polymer (PIDT) of 1.66 eV while the bottom subcell contained a narrow bandgap polymer (PY-IT) of 1.40 eV. The two subcells employed polymer donors of PM7 in the top subcell and PM6 in the bottom subcell. The best PCE for this fabricated tandem was 17.87%, employing the structure ITO/PEDOT:PSS/PM7:PIDT/C_60_/BPhen/PEDOT:PSS/PM6:PY-IT/PDINN/Ag [16].

Apart from this, indoor PVs have emerged as a promising solution for powering wireless Internet of Things (IoT) devices without relying on batteries as the use of batteries is unsustainable and cost ineffective. By harvesting indoor light energy, IoT devices can operate for long periods of time without requiring costly maintenance or replacements. PV devices based on various materials such as silicon, organic and polymer semiconductors, CIGS, and perovskites have been tested for indoor applications, with single active layer devices showing high PCEs under light-emitting diode (LED) lighting [22,23,24,25,26,27]. Although single active layer indoor PVs have shown high efficiencies under LED lighting, tandem structures have the potential to further increase the PCE and stability of indoor PVs. However, very few efforts have been devoted to studying tandem cells under LED illumination [28]. Therefore, tandem structures may be a promising area of research for indoor PVs, and further studies are needed to optimize their performance for indoor applications.

In this work, the white LED illumination impact on tandem all-PSCs was investigated for the first time by employing TCAD simulations based on the Silvaco Atlas device simulator. In addition, the results of the tandem are compared with both the wide bandgap front and narrow bandgap rear subcells. First, the simulation results with experimental studies were validated for ensuring the reliability and verification of the simulation technique and the assumptions made in the model. The tandem utilized in this study is based on a previously published work regarding all-PSCs for both top and bottom subcells [16]. Next, the two subcells and tandem under the LED lightening were investigated for different color temperatures and intensities. The simulation results showed a superior performance for the wide bandgap front subcell. On the other hand, the tandem performance was found to deteriorate. Thus, a proposed design was introduced to optimize the tandem for use in indoor applications.

## 2. Materials and Methods

### 2.1. Solar Cell Configurations and Material Parameters

**Figure 1:**
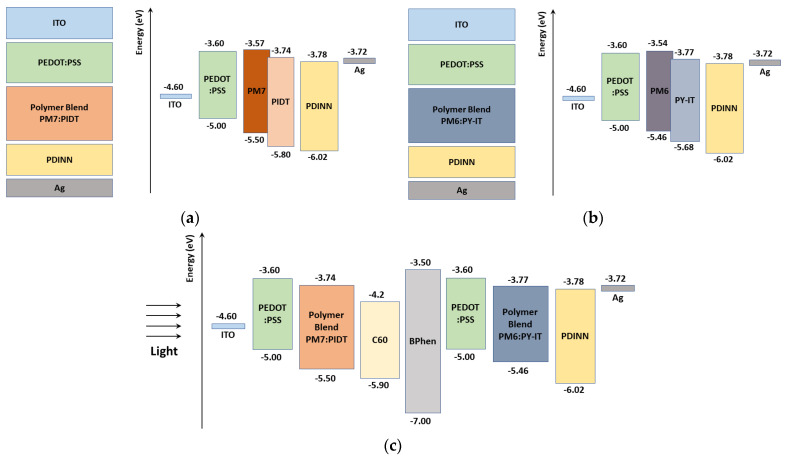
Basic layer structures and energy band diagrams before contact for the (**a**) front subcell, (**b**) rear subcell, and (**c**) tandem cell.

The tandem cell under investigation comprises all-PSCs for the front and the rear subcells. It is based on an experimental cell as published in [16]. The traditional device structure of ITO/PEDOT:PSS/Blend (PM7:PIDT for the top cell and PM6:PY-IT for the bottom cell)/PDINN/Ag was used to fabricate the standalone all-PSCs, where ITO-coated glass substrates were utilized. The device structures and energy level diagrams of the standalone all-PSCs with PM7:PIDT top cell and PM6:PY-IT bottom cell are shown in Figure 1a,b, respectively. The *E*_HOMO_ and *E*_LUMO_ energy levels of the two PIDT and PY-IT were determined to be −5.80/−5.68 and −3.74/−3.77 eV, respectively, through electrochemical cyclic voltammetry, while the mobilities were measured using the photo-CELIV technique [16]. Further, the absorption spectra of both PM7:PIDT and PM6:PYIT blend active layers showed a good complementary in the 300–900 nm wavelength band. Some typical parameters, extracted from the literature as indicated, of the different layers of all-PSCs are listed in Table 1 [16,29,30,31]. PEDOT:PSS and PDINN (or C60) act as a hole transport layer (HTL) and electron transport layer (ETL), respectively. The work functions of both ITO and Ag contacts are 4.7 and 3.72 eV, respectively [30,31]. Regarding the tandem device, the ETL of the front subcell is taken to be C60 instead of PDINN utilized in the standalone cells. The energy band profile of the tandem is displayed in Figure 1c, where BPhen serves as an interlayer.

### 2.2. Simulation Methodology

**Figure 2:**
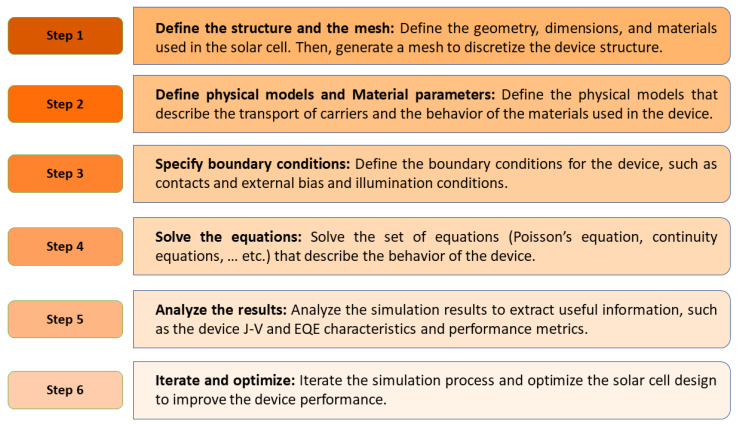
Basic TCAD simulation steps to simulate and optimize a certain solar cell structure.

For the current simulation study, the design and evaluation of the tandem as well as the standalone all-PSCs were carried out using the Silavco Atlas simulator. This TCAD software is widely used in modeling and simulating thin-film solar cells [32]. In this simulator, Poisson’s equation is solved concurrently with electron and hole continuity equations. Using the drift-diffusion transport model, the electron and hole current densities are then determined. It is important to note that the recombination mechanisms considered include Shockley–Read–Hall (SRH), direct band-to-band, and Auger recombination. Additionally, the definitions of defect properties such as trap energy levels and densities can be incorporated in the bulk of the material or at the interfacial zones. The boundary conditions of front and back contacts are set to accomplish thermionic emission, with identified surface recombination velocities for both electrons and holes. Further, a lumped resistance is included as an interlayer instead of the BPhen interlayer in the following simulation, which allows the current to flow freely between the two subcells of the tandem device without significant impedance. Regarding the optical simulation, the absorption coefficients are extracted from experimental data and are then fine-tuned to match the measured external quantum efficiency (EQE), resulting in a correct estimation for the short circuit current density. A general outline of the simulation process and the specific steps can be summarized in a flowchart as can be depicted in Figure 2.

### 2.3. Calibration of Standalone and Tandem Cells

**Figure 3:**
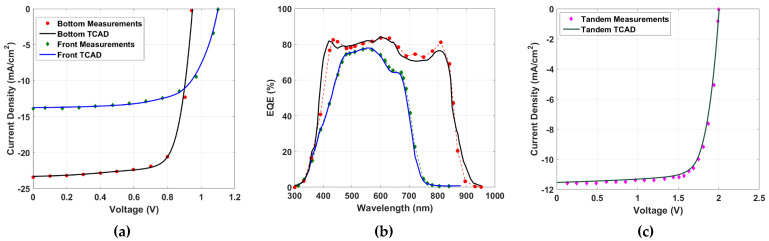
Calibration of simulated vs. measured data: (**a**) *J*-V curves for both subcells, (**b**) EQE spectra for both subcells, and (**c**) *J-V* for the tandem all-PSCs.

To ensure the accuracy of our simulation, we performed a calibration step to validate the model implemented in the Atlas simulator. The *J-V* (current density-voltage) curves for the top and bottom subcells, obtained from the simulation and experimental data extracted from [16], are presented in Figure 3a, along with the simulated EQEs presented in Figure 3b. The PV parameters obtained from the simulation are listed in Table 2, where *J_SC_* is the short-circuit current density and *V_OC_* is the open-circuit voltage, while FF represents the fill factor. As indicated in the table, the output parameters matched well with the experimental results. The results revealed that the front cell had a lower PCE than that of the rear cell for these designed cells. Furthermore, the tandem cell was simulated using the same layers encountered in the experimental work. The *J-V* characteristics are exhibited in Figure 3c, while the PV factors are demonstrated in Table 2, signifying a decent fit for the simulation compared to the measurements and emphasizing the validation of the TCAD models. It should be pointed out here that the optical bandgaps of the blends were different from the electrical bandgap values (that can be deduced from Table 1). From the EQE curves, the estimated values of the optical energy gaps for the front and rear blends were 1.55 eV and 1.3 eV, respectively. Therefore, the two blends were complementary and can cover a wide range of the spectrum.

### 2.4. Indoor Light LED Characteristics

**Figure 4:**
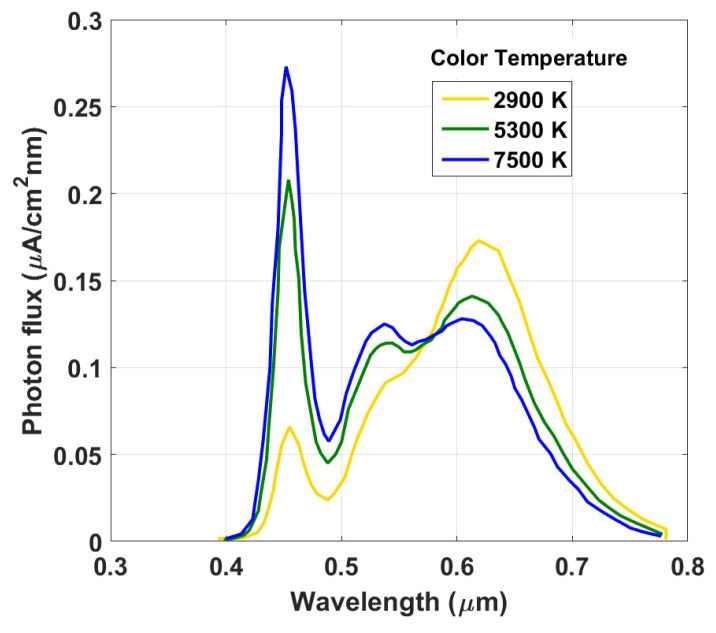
White LED photon flux variation with different color temperatures as a function of wavelength.

The emission spectra of commonly used indoor light sources, including fluorescent lamps and LEDs, typically cover a range of 400 to 700 nm with intensities below 1 mW/cm^2^ [33]. These spectra are much narrower and have weaker intensities than the standard AM1.5G spectrum. This means that PV cells developed for converting sunlight may not be optimally suited for indoor applications. In addition, the solar cell response according to indoor light sources is completely different from that under AM1.5G illumination. Thus, to assess the performance of all-PSCs presented herein under the influence of indoor light source, we used the spectra of artificial white LEDs, which consist of a sharp blue emission from GaN. The illuminance of the incident light can be adjusted from 200 to 10,000 lx (which corresponds to 57.9–2895 μW/cm^2^) [33]. Figure 4 shows the photon flux as a function of wavelength for incident LED light sources with varying color temperatures, as extracted from [33]. A temperature of 7500 K corresponded to a cool white LED, where the photon flux peaked at around 450 nm in the blue part of the spectrum. The flux gradually decreased as the wavelength increased towards the red part of the spectrum. For a color temperature of 5300 K, the photon flux had another slight peak at around 630 nm, which was in the green-yellow part of the spectrum. A warm white LED occurred for 2900 K, wherein the photon flux peaked at around 630 nm, which was in the orange-red part of the spectrum. The flux gradually decreased as the wavelength increased towards the blue part of the spectrum.

## 3. Results and Discussions

This section presents simulation results for the behavior of standalone and tandem all-PSCs under different conditions. The first step involved investigating the impact of the color temperature of indoor white LED light on the PV parameters of the cells. Next, the influence of intensity was introduced at a temperature of 2900 K. For each step, the PV parameters of the top, bottom, and tandem cells are listed and compared to assess their performance. Finally, a proposed design is presented to optimize the performance of the tandem cell under the influence of white LED lighting.

### 3.1. Impact of LED Color Temperature

**Figure 5:**
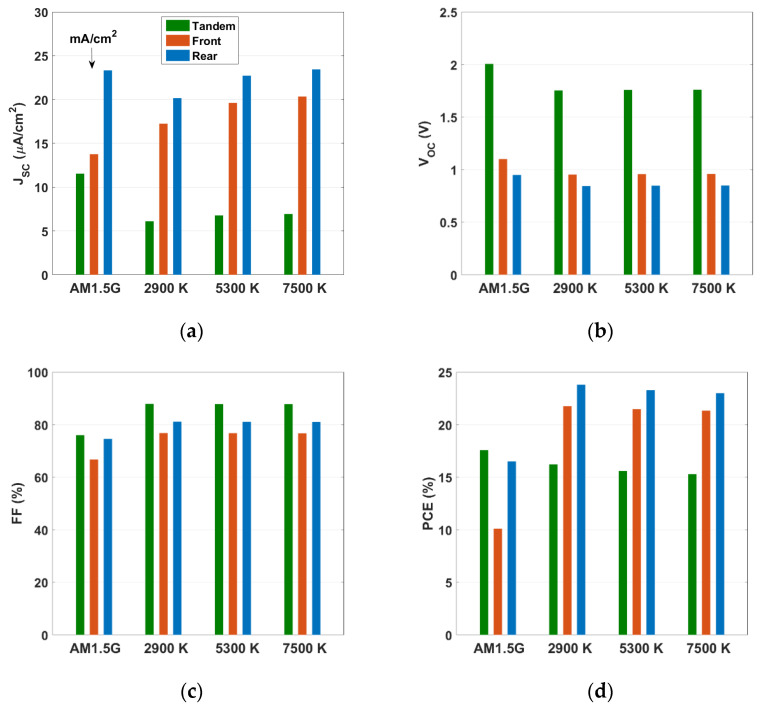
Variation of PV parameters of the top, bottom, and tandem all-PSCs for different values of LED color temperature. The values at the AM1.5G illumination are also shown for comparison. (**a**) *J_SC_* (noting that the units for AM1.5G are mA/cm^2^ while that for LED illumination are μA/cm^2^), (**b**) *V_OC_*, (**c**) FF, and (**d**) PCE.

To verify the behavior of the solar cells under investigation with respect to LED color temperature, the indoor performances of the three all-PSC devices were assessed by selecting temperatures of 2900, 5300, and 7500 K. Figure 5 displays the PV metrics for the top (wide bandgap), bottom (narrow bandgap), and tandem cells. The metrics related to the AM1.5G illumination are also shown for comparison. The *J_SC_* behavior, seen in Figure 5a, indicates an enhancement when increasing the temperature for the front subcell. The reason behind this is that the front subcell had a higher propensity to capture photons from the longer end of the visible spectrum (see Figure 3b) as the cutoff wavelength of the front subcell was about 0.8 μm, resulting in optical responses that were more compatible with the spectrum of the white LED (see Figure 4). In addition, the *J_SC_* obtained under LED illumination was lower than that obtained under AM1.5G illumination, due to the reduced light intensity of the LED illumination compared to AM1.5G.

Regarding the trend of *V_OC_* for the front subcell (as depicted in Figure 5b), *V_OC_* was nearly independent of the color temperature, while its values under the influence of indoor light were slightly lower than that under AM1.5G. The reason for the decline of *V_OC_* values under LED illumination than AM1.5G illumination can be explained based on the following approximate analytical expression [34],

(1) $$V_{OC}\approx nV_{T}\ln\left(\frac{J_{SC}}{J_{o}}\right)$$

where the ideality factor and thermal voltage are denoted as *n* and *V_T_*, respectively, while *J_o_* is the reverse saturation current density. As *J_SC_* decreases with the reduction in the incident light intensity (see Figure 5a), *V_OC_* decreases as well according to Equation (1). Consequently, the values of *V_OC_* under AM1.5G are higher than those under LED illumination. The same trend is valid for the FF, except that the values of FF when involving LED are higher than the value of FF for AM1.5G. The overall consequence is that the PCE was nearly independent of the color temperature of the LED light, while it was much higher than the initial value recorded under AM1.5G. For instance, at 2900 K, the obtained PCE was 21.757%, while its value was 10.110% under 1-sun illumination.

Furthermore, when considering the rear subcell, it can be inferred from Figure 5a that the *J_SC_* increased with temperature. However, the *J_SC_* values obtained under indoor lighting conditions were slightly lower than those obtained under AM1.5G, which can be attributed to the optical response of the rear cell that extended to around 950 nm (see Figure 3b). This means that it was more optimized for AM1.5G than for white LED illumination. The same patterns were observed in the *V_OC_* and FF of the rear cell as in the front cell. Additionally, the PCE slightly increased with temperature, as demonstrated in Figure 5d, with values that were higher than the PCE at AM1.5G (the PCE value at 2900 K was about 23.794%, while the calibrated value was 16.504%). However, the rate of increase in efficiency was lower than that of the front cell.

On the other hand, Figure 5a shows that the situation was different for the tandem cell. Although the *J_SC_* increased with color temperature, its values were still significantly lower than the *J_SC_* at AM1.5G. This was an expected trend because the tandem cell was not optimized for the current matching point under indoor lighting conditions, resulting in a current mismatch between the two subcells that made up the tandem. Moreover, the *V_OC_* trend was similar to that of the two subcells, as it was almost equal to the sum of the individual *V_OC_* of each subcell. The FF also exhibited similar behavior to the two subcells, as it was almost independent of LED temperature. However, the values of FF obtained under LED illumination were higher than those obtained under AM1.5G. This can be explained by the fact that as the light intensity decreased, the *J_SC_* also decreased, resulting in a lower loss in the FF, as mentioned in REF [35]. Overall, the PCE of the tandem cell slightly decreased with temperature, with values (16.220% at 2900 K) lower than the initial tandem under AM1.5G (17.578%).

To gain insight into the most dominant factor that affects the PCE, one can use the expression [36],

(2) $$\mathrm{PCE}\left(\%\right)=\frac{J_{SC}V_{OC}\mathrm{FF}}{P_{\mathrm{in}}}\times 100$$

where P_in_ is the input power (W/cm^2^). According to the results presented in Figure 5, it was observed that changes in the FF and *V_OC_* were insignificant, whereas variations in the *J_SC_* were more prominent. This suggests that *J_SC_*, which was directly linked to the optical response of the photoactive layer, was the key factor that had a direct impact on the PCE. Moreover, to demonstrate the relative change in the PCE when applying indoor LED light instead of the AM1.5G illumination, we define a relative percentage factor Δζ, where

(3) $$\Delta \zeta \left(\%\right)=\frac{{\mathrm{PCE}}_{\mathrm{indoor}}-{\mathrm{PCE}}_{\mathrm{AM}1.5G}}{{\mathrm{PCE}}_{\mathrm{AM}1.5G}}\times 100$$

**Figure 6:**
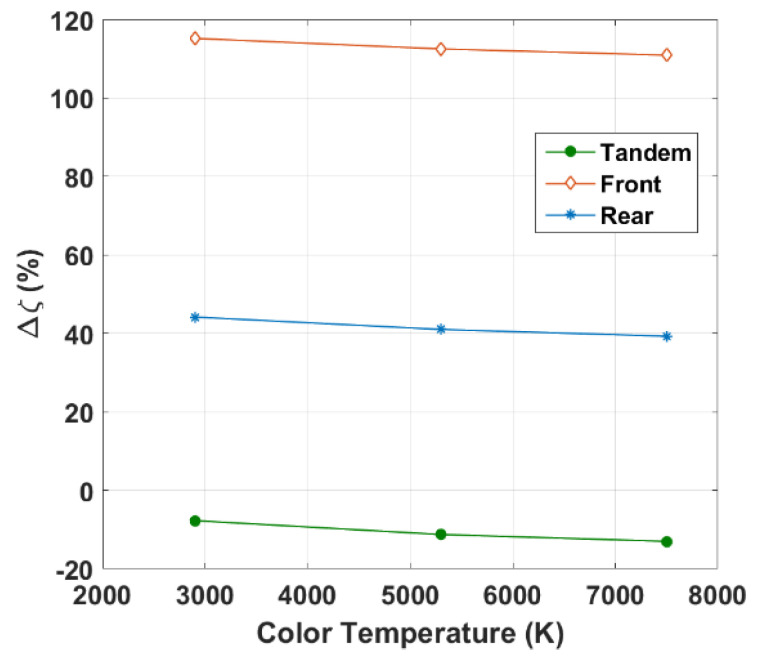
Variation of relative PCE percentage against LED color temperature for top, bottom, and tandem all-PSCs.

Figure 6 displays Δζ against the color temperature for the three cases (top, bottom, and tandem cells). As indicated in the figure, the front cell showed the highest relative PCE percentage, where Δζ = 115.2% at 2900 K. Meanwhile, the tandem demonstrated negative values, implying a performance degradation when applied to indoor conditions.

### 3.2. Impact of LED Intensity

**Figure 7:**
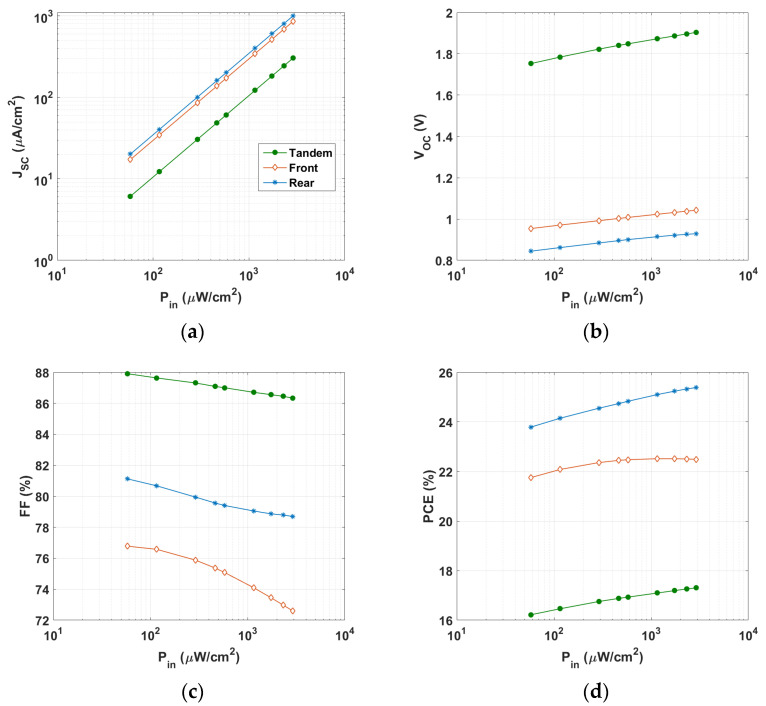
Variation of PV parameters of the top, bottom, and tandem all-PSCs for different values of LED illumination intensity: (**a**) *J_SC_*, (**b**) *V_OC_*, (**c**) FF, and (**d**) PCE.

**Figure 8:**
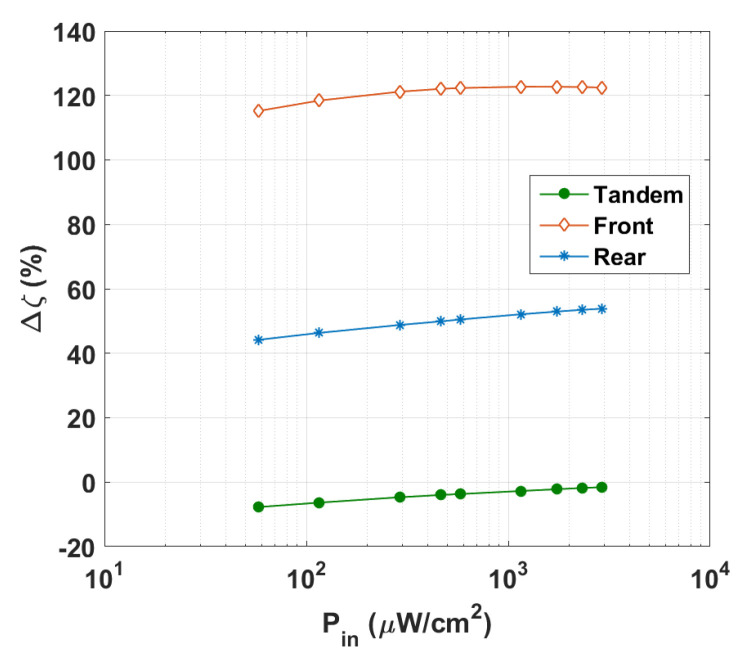
Variation of relative PCE percentage against LED input power for top, bottom, and tandem all-PSCs.

Next, as demonstrated in Figure 7, the changes of the PV metrics as a function of the incident LED light intensity were further explored by varying its input power in the range 57.9 to 2895 μW/cm^2^ (which corresponded to illuminance in the range of 200–10,000 lx) at a constant color temperature of 2900 K. As depicted in Figure 7a, the *J_SC_* values of the three cells were almost proportional to P_in_, which was confirmed with the experimental results [37]. This linear relationship indicated that bimolecular charge recombination, a process where two free charge carriers combine and recombine, is effectively suppressed under short-circuit conditions. Additionally, when increasing the input power, the *V_OC_* values of the three cells increased over the whole range (see Figure 7b). Meanwhile, the FF values decreased with P_in_ (see Figure 7c). On the other hand, the PCE trend of the three cells was not alike. While the PCE of the back and tandem cells increased with input power, the behavior of the front subcell was different. The PCE for the front cell increased up to a certain limit and then nearly saturated, with a maximum value of 22.52% at P_in_ = 1160 μW/cm^2^. Furthermore, Figure 8 shows Δζ against P_in_ for the three cells. As shown in the figure, the front cell exhibited the highest relative PCE percentage.

### 3.3. Optimization of Tandem Cells for an Indoor Environment

Based on the previous simulation results, it has been revealed that the tandem cell is not suitable for indoor conditions as it is optimized for the outdoor one-sun environment. Therefore, it should be specifically optimized for indoor applications to be employed in low-intensity circumstances. Thus, in order to enhance the indoor tandem cell capabilities, a current matching condition should be engineered to achieve maximum PCE. For this purpose, we conducted a simulation run in which both top and bottom polymer blend thicknesses were varied concurrently to search for the optimum efficiency. The range of the top thickness (*t_top_*) and bottom thickness (*t_bot_*) was taken to be from 30 to 100 nm and from 100 to 200 nm, respectively.

**Figure 9:**
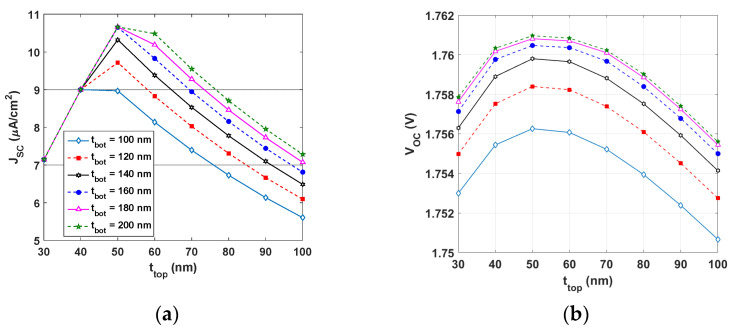

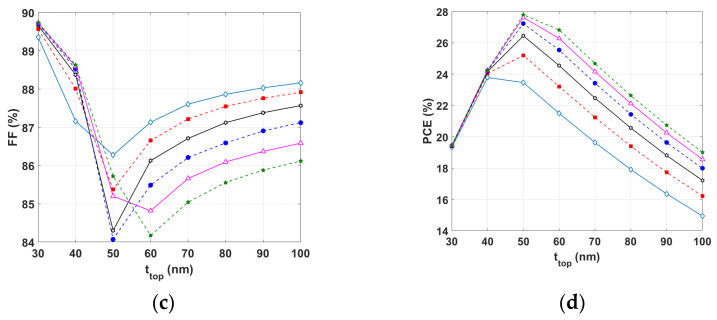
Variation of PV parameters of tandem all-PSCs for different values of top and bottom subcell thicknesses (at 2900 K and 200 lx): (**a**) *J_SC_*, (**b**) *V_OC_*, (**c**) FF, and (**d**) PCE.

Figure 9 represents the PV metrics of the tandem all-PSC under a warm LED condition (at 2900 K) and 200 lx. It can be observed from Figure 9a that *J_SC_* was maximum for *t_top_* = 50 nm (which was half the initial value) as long as *t_bot_* was higher than 160 nm. The maximum *J_SC_* was about 10.66 μA/cm^2^. Moreover, *V_OC_* had a local maximum at *t_top_* = 50 nm for any value of *t_bot_* (see Figure 9b). The situation was different for the FF, as displayed in Figure 9c; however, the minimum available value was 84%. The trends of *J_SC_*, *V_OC_*, and FF reflected clearly on the PCE shown in Figure 9d, where the maximum PCE occurred for *t_top_* = 50 nm, keeping the bottom thickness higher than 160 nm. On the basis of the simulation results, using a *t_bot_* of 200 nm resulted in an optimal PCE of approximately 27.80%.

Thus, to obtain higher PCE values under white LED lighting, it is recommended to increase the bottom thickness while decreasing the top thickness. It should be mentioned that increasing the thickness of the bottom layer will result in a higher PCE, but this comes at the price of increased processing complexity and costs. These interesting findings highlight the importance of optimizing the thicknesses of both the top and bottom cells in order to enable the tandem cell to perform effectively under indoor conditions.

## 4. Conclusions

In this TCAD simulation study, the performance of all-PSCs based on two complementary polymer blends, PM7:PIDT and PM6:PYIT, as well as the tandem produced from integrating them, was assessed. The two standalone PSCs were evaluated under both outdoor (AM1.5G) and indoor light (white GaN LED) environments. Additionally, the tandem cell was evaluated under the same criteria to provide a fair comparison. On the basis of simulations, it was found that the tandem device had a higher PCE than that of the standalone cells for AM1.5G illumination, as verified experimentally. Yet, upon the illumination of white LED for different color temperatures, the behavior was completely different. The front cell, which had a wider bandgap, was found to provide the highest relative PCE percentage, while the rear cell, which had a narrower bandgap, achieved higher efficiencies than the PCE under AM1.5G; however, its relative PCE percentages were lower than those obtained from the top cell. The PCE of the front cell was increased from the calibrated value of 10.110% to 21.757% at 2900 K.

On the other hand, when comparing the performance of the tandem cell with the two constituting subcells, a deterioration in the performance was observed. The PCE was found to be 16.220% at 2900 K instead of the calibrated value of 17.578% (under one sun). This can be attributed to the low *J_SC_* of the tandem cell in the indoor light condition. Thus, a tandem device with complementary band gaps and thicknesses optimized for outdoor conditions is not compatible to be utilized in operation under white LEDs with low light intensities. To address this challenge, we suggest designing the tandem cell for indoor lighting specifically by carefully selecting the thicknesses of the top and bottom absorber layers to achieve the current matching. A potential solution would involve decreasing the thickness of the top absorber layer while slightly increasing the bottom absorber layer thickness. This approach can result in a higher PCE of 27.80% at a color LED temperature of 2900 K. The proposed approach of designing tandem structures for indoor lighting can highlight the potential for further improving the PCE and stability of indoor PVs.

## Figures and Tables

**Table 1:** Key material parameters of the various layers of tandem all-PSCs.

Parameters	PEDOT:PSS [29]	PM7:PIDT [16]	PM6:PYIT [16]	PDINN [30]	C60 [31]
*t* (nm)	30	100	100	30	20
*E*_LUMO_ (eV)	3.40	3.74	3.77	3.78	4.2
*E*_HOMO_ (eV)	5.00	5.50	5.46	6.02	5.9
*ε_r_*	3.0	3.0	3.0	5.0	5.0
*µ_n_* (cm^2^/V·s)	4.5 × 10^−4^	2.89 × 10^−4^	1.97 × 10^−4^	2 × 10^−6^	0.08
*µ_p_* (cm^2^/V·s)	9.9 × 10^−5^	2.89 × 10^−4^	1.97 × 10^−4^	1 × 10^−3^	0.0035
*N_c_* (cm^−3^)	1 × 10^22^	1 × 10^21^	1 × 10^21^	1 × 10^19^	2.2 × 10^18^
*N_v_* (cm^−3^)	1 × 10^22^	1 × 10^21^	1 × 10^21^	1 × 10^19^	1.8 × 10^19^
*N_D_* (cm^−3^)	-	-	-	1 × 10^19^	1 × 10^17^
*N_A_* (cm^−3^)	5 × 10^19^	-	-	-	-

**Table 2:** Key PV metrics extracted from TCAD and experimental illuminated *J-V* characteristics for both front and rear subcells. All experimental data were extracted from [16].

PV Parameters		*J*_SC_ (mA/cm^2^)	*V*_OC_ (V)	*FF* (%)	*η* (%)
Front subcell	Exp.	14.0 ± 0.3	1.10 ± 0.01	65.3 ± 1.2	10.1 ± 1.3
	TCAD	13.764	1.101	66.768	10.110
Rear subcell	Exp.	23.2 ± 0.6	0.94 ± 0.01	72.6 ± 1.5	16.0 ± 0.4
	TCAD	23.330	0.949	74.584	16.504
Tandem cell	Exp.	11.5 ± 0.2	2.00 ± 0.01	75.0 ± 1.2	17.6 ± 0.2
	TCAD	11.543	2.005	75.975	17.578

## Data Availability

Not applicable.
